# Citrus Flavanones Affect Hepatic Fatty Acid Oxidation in Rats by Acting as Prooxidant Agents

**DOI:** 10.1155/2013/342973

**Published:** 2013-10-31

**Authors:** Rodrigo Polimeni Constantin, Gilson Soares do Nascimento, Renato Polimeni Constantin, Clairce Luzia Salgueiro, Adelar Bracht, Emy Luiza Ishii-Iwamoto, Nair Seiko Yamamoto, Jorgete Constantin

**Affiliations:** ^1^Department of Biochemistry, Laboratory of Liver Metabolism, University of Maringá, 87020900 Maringá, PR, Brazil; ^2^Department of Physiological Sciences, University of Maringá, 87020900 Maringá, PR, Brazil

## Abstract

Citrus flavonoids have a wide range of biological activities and positive health effects on mammalian cells because of their antioxidant properties. However, they also act as prooxidants and thus may interfere with metabolic pathways. The purpose of this work was to evaluate the effects of three citrus flavanones, hesperidin, hesperetin, and naringenin, on several parameters linked to fatty acid oxidation in mitochondria, peroxisomes, and perfused livers of rats. When exogenous octanoate was used as substrate, hesperetin and naringenin reduced the mitochondrial NADH/NAD^+^ ratio and stimulated the citric acid cycle without significant changes on oxygen uptake or ketogenesis. When fatty acid oxidation from endogenous sources was evaluated, hesperetin and naringenin strongly reduced the mitochondrial NADH/NAD^+^ ratio. They also inhibited both oxygen uptake and ketogenesis and stimulated the citric acid cycle. Hesperidin, on the other hand, had little to no effect on these parameters. These results confirm the hypothesis that citrus flavanones are able to induce a more oxidised state in liver cells, altering parameters related to hepatic fatty acid oxidation. The prooxidant effect is most likely a consequence of the ability of these substances to oxidise NADH upon production of phenoxyl radicals in the presence of peroxidases and hydrogen peroxide.

## 1. Introduction

Citrus flavonoids are found at high concentrations in citrus fruits; the most common classes are flavanones, flavones, and flavonols [1]. These compounds exhibit a wide range of biological activities and positive health effects on mammalian cells, including antiinflammatory, antiatherogenic, and anticancer effects. The antioxidant properties of citrus flavonoids and their metabolites have been found to be at least partially responsible for these therapeutic actions [2–4].

**Figure 1:**
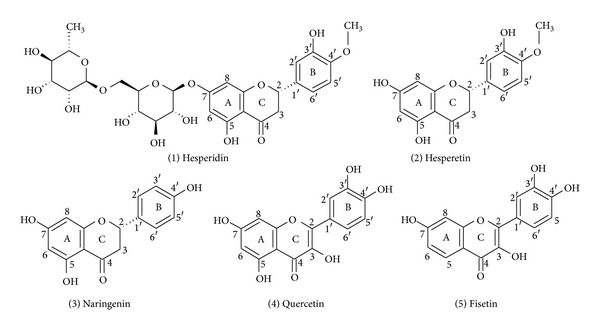
Chemical structures of the flavanones hesperidin (1), hesperetin (2), and naringenin (3) and of the flavonols quercetin (4) and fisetin (5). The rings of each compound are highlighted.

The antioxidant activities of citrus flavonoids and their metabolites depend upon their molecular structures. The arrangements of hydroxyl, methoxy, and glycosidic side groups, as well as the conjugation between the aromatic rings are important features that govern antioxidant activity [5]. For example, the antioxidant capacities of quercetin and fisetin, two abundant dietary flavonols, are favoured by some structural peculiarities (Figure 1): (a) a high degree of hydroxylation; (b) presence of 2,3 unsaturation in conjugation with the 4-oxo group in the C-ring; (c) a dihydroxylated B-ring (catechol), which allows prompt hydrogen donation (electrons); and (d) the presence of both 3- and 5-hydroxyls in quercetin and 3-hydroxyls in fisetin [6–10]. However, not all studies confirm the antioxidant effects of quercetin, fisetin and other flavonoids, and several report that they have prooxidant capabilities [11–13]. Both quercetin and fisetin are able to promote NADH oxidation because of interactions with cellular enzymes and their abilities to shift cellular conditions to a more oxidised state [12, 13]. This state is reflected by changes in several parameters of cellular metabolism, such as inhibition of gluconeogenesis and ketogenesis [12–16].

This prooxidant effect is apparently not restricted to quercetin and fisetin; indeed, previous studies have shown that citrus flavonoids belonging to the flavanones, for example, hesperidin, hesperetin, and naringenin (Figure 1), can form prooxidant metabolites that oxidise NADH upon oxidation by peroxidase/H_2_O_2_ [17, 18]. Despite obvious structural differences between flavonols and flavanones (e.g., flavanones are nonplanar, have a chiral centre at C2, and contain a saturated C2-C3 bond and a phenol B ring), they could have effects on liver metabolism similar to those caused by the flavonols quercetin and fisetin [12, 13].

The main purpose of this work was to assess whether three common flavanones, namely, hesperidin, hesperetin, and naringenin (Figure 1), have prooxidant activities in liver cells and determine in the effects of their differing structures on various metabolic parameters. For this study, we used isolated perfused rat liver and subcellular liver fractions. 

A more detailed characterisation of the mode of action of citrus flavanones in mammalian cells (especially that of any potential prooxidant effects) is important because these substances have generally been considered to be effective antioxidants that are beneficial in the contexts of cancer, inflammation, and atherosclerosis [2–4].

## 2. Materials and Methods 

### 2.1. Materials

Hesperidin, hesperetin, naringenin, NADH, NAD^+^, succinate, L-malate, L-carnitine, palmitoyl-L-carnitine, octanoyl-L-carnitine, octanoyl-CoA, palmitoyl-CoA, 2′,7′-dichlorofluorescein diacetate (DCFH-DA), 2′,7′-dichlorofluorescein (DCF), 2,4-dinitrophenol (DNP), 2,5-diphenyloxazole, 2,2-p-phenylenebis(5-phenyloxazole), fatty acid-free serum albumin, phenylmethylsulfonyl fluoride (PMSF), horseradish peroxidase (HRP) type VI-A, D-*β*-hydroxybutyrate, D-*β*-hydroxybutyrate dehydrogenase from *Pseudomonas lemoignei*, and labelled octanoate ([1-^14^C]octanoate), (50 *μ*Ci/mmol) were purchased from Sigma-Aldrich Chemical Co. (St. Louis, USA). All other chemicals were of the best available grade.

### 2.2. Animals

Male Wistar rats weighing 180–220 g and fed *ad libitum* with a standard laboratory diet (Nuvilab CR-1) were housed in polycarbonate cages in a controlled environment, with a 12 : 12 h light-dark cycle starting at 06:00 AM, at 20–23°C. Overnight-fasted animals were used in all experimental protocols. All experiments were conducted in strict adherence with the guidelines of the Ethics Committee for Animal Experiments of the University of Maringá (CEAE, Registered number 014/2009, Protocol 002/2009), which is in accordance with the internationally accepted recommendations for the care and use of animals.

### 2.3. Liver Perfusion

The liver perfusion apparatus was built in the workshops of the University of Maringá. For the surgical procedure, the animals were anaesthetised by intraperitoneal injection of sodium pentobarbital (50 mg/kg). The criterion for anaesthesia was lack of body or limb movement in response to a standardised tail clamp stimulus. The haemoglobin-free, nonrecirculating perfusion was performed according to the technique described by Scholz and Bücher [19]. After cannulation of the portal and cava veins, the liver was positioned in a plexiglass chamber. The hepatic artery was closed (monovascular perfusion) and the bile duct left open. Flow was maintained at a constant rate using a peristaltic pump (Miniplus 3, Gilson, France) and was adjusted to between 30 and 32 mL/min, depending on the liver weight. The perfusion fluid was Krebs/Henseleit-bicarbonate buffer (pH 7.4) containing 25 mg% (w/v) bovine serum albumin. It was saturated with a mixture of oxygen and carbon dioxide (95 : 5) using a membrane oxygenator with a simultaneous temperature adjustment to 37°C. The composition of the Krebs/Henseleit-bicarbonate buffer was as follows: 115 mM NaCl, 25 mM NaHCO_3_, 5.8 mM KCl, 1.2 mM Na_2_SO_4_, 1.18 mM MgCl_2_, 1.2 mM NaH_2_PO_4_, and 2.5 mM CaCl_2_. After the stabilization of oxygen consumption the experiments were initiated and samples of the effluent fluid were collected at time intervals of 2 minutes. In some experiments, substrate-free perfusion fluid was used, and trace amounts of [1-^14^C]octanoate (0.01 *μ*Ci/mL) were infused from the beginning of the perfusion experiments. In other experiments, a mixture of octanoate (0.2 mM) and [1-^14^C]octanoate (0.01 *μ*Ci/mL) was infused starting at 10 minutes of perfusion. According to Soboll et al. [20], this procedure effectively measures the citric acid cycle via labelling of acetyl-CoA. Consequently, ^14^CO_2_ production can be regarded as an indicator of citric acid cycle activity. These substrates were infused for a defined time period, according to experimental protocol, and this period corresponds to a control condition. Subsequently, the citrus flavanones were infused dissolved in the same preceding perfusion fluid. The solubilisation of these compounds was achieved by the simultaneous addition of an equivalent amount of 1.0 M NaOH. After this procedure, the pH of the perfusion fluid containing the flavanones was adjusted to 7.4 before use. Samples of the effluent perfusion fluid were collected at 2 min intervals and analysed for their metabolite content. Acetoacetate and *β*-hydroxybutyrate were assayed by means of standard enzymatic procedures using *β*-hydroxybutyrate dehydrogenase [21]. Interference by citrus flavanones (absorbance at 340 nm) was excluded by running blanks. The oxygen concentration in the outflowing perfusate was continuously monitored by employing a Teflon-shielded platinum electrode adequately positioned in a plexiglass chamber where the perfusate exited [22]. The carbon dioxide production from [1-^14^C]octanoate was measured by trapping ^14^CO_2_ in phenylethylamine [23]. Radioactivity was measured by liquid scintillation spectroscopy. The following scintillation solution was used: toluene/ethanol (2/1) containing 5 g/L 2,5-diphenyloxazole and 0.15 g/L 2,2-p-phenylenebis(5-phenyloxazole). Metabolic rates were calculated from the differences between the input and output and the total flow rates and were analysed in reference to the wet weights of the livers. 

### 2.4. Isolation of Mitochondria and Peroxisomes

The livers were removed and cut into small pieces. These fragments were suspended in a medium containing 0.2 M mannitol, 75 mM sucrose, 1.0 mM tris(hydroxymethyl)aminomethane hydrochloride (Tris-HCl, pH 7.4), 1.0 mM ethylene glycol-bis(2-aminoethylether)-N,N,N′,N′-tetraacetic acid (EGTA), 0.1 mM phenylmethanesulfonyl fluoride (PMSF), and 50 mg% (w/v) fatty acid-free bovine serum albumin. Homogenisation was carried out in the same medium by means of a Dounce homogeniser. After homogenisation, the mitochondria were isolated by differential centrifugation according to Voss et al. [24] and suspended in the same medium (protein concentration of 70–80 mg/mL), which was kept at 0–4°C. 

Peroxisomes were isolated according to the method described by Natarajan et al. [25]. The livers were removed and cut into small pieces. These fragments were suspended in a medium containing 230 mM mannitol, 70 mM sucrose, 1 mM EDTA (ethylenediaminetetraacetic acid), 0.1 mM PMSF, and 3 mM 4-(2-hydroxyethyl)piperazine-1-ethanesulfonic acid (HEPES), pH 7.4. Homogenisation was carried out in the same medium by means of a Dounce homogeniser. Peroxisomes were isolated by differential centrifugation. The homogenate was first centrifuged at 600 g for 10 minutes to remove cell debris, and mitochondria were pelleted by centrifugation at 15,000 g for 5 minutes. The postmitochondrial supernatant was then centrifuged at 39,000 g for 10 minutes to isolate the fraction containing peroxisomes, which was resuspended in 250 mM sucrose containing 1.0 mM EDTA, 0.1 mM PMSF, and 10 mM Tris-HCl (pH 7.3) and homogenised using a Dounce homogeniser. This suspension was centrifuged at 15,000 g for 10 minutes to remove mitochondrial contamination. Afterwards, the supernatant was again centrifuged at 39,000 g for 10 minutes to isolate the peroxisomes, which were resuspended, homogenised, and adjusted to a final protein concentration of approximately 1.0 mg/mL.

### 2.5. Determination of Oxygen Uptake by Isolated Mitochondria Oxidising Fatty Acids

Oxygen uptake by isolated mitochondria oxidising fatty acids was measured polarographically using a Teflon-shielded platinum electrode [22–24]. The incubation medium contained 2.0 mM KH_2_PO_4_, 10 mM 4-(2-hydroxyethyl)piperazine-1-ethanesulfonic acid (HEPES, pH 7.2), 0.1 mM EGTA, 130 mM KCl, 5.0 mM MgCl_2_, 0.1 mM 2,4-dinitrophenol (2,4-DNP), 2.5 mM L-malate, and 50 mg% (w/v) fatty acid-free bovine serum albumin [26]. Mitochondria (0.6–1.2 mg protein/mL) were incubated in final volumes of 2.0 mL. The reaction was initiated by the addition of (a) 20 *μ*M octanoyl-CoA + 2.0 mM L-carnitine, (b) 20 *μ*M octanoyl-L-carnitine, (c) 20 *μ*M palmitoyl-CoA + 2.0 mM L-carnitine, or (d) 20 *μ*M palmitoyl-L-carnitine. In the control series, oxygen uptake was followed by nearly five minutes and the rates of oxygen uptake were computed from the slopes of the recorder tracings. The citrus flavanones at final concentrations varying from 10 to 300 *μ*M (dissolved in 0.1 M dimethylformamide) were added to the incubation medium, two minutes before the substrates. Control experiments were performed to exclude solvent effects. The data were expressed as nmol of O_2_ consumed per min per mg of mitochondrial protein.

### 2.6. Mitochondrial Membrane-Bound Enzymatic Activities

Rat liver mitochondria, isolated as described above [24], were disrupted by successive freeze and thawing procedures using liquid nitrogen and used as an enzyme source for assaying membrane-bound enzymatic activities. NADH-oxidase and succinate-oxidase activities were assayed polarographically using a 20 mM Tris-HCl (pH 7.4) medium. The reactions were started by the addition of 1.0 mM NADH or 10 mM succinate [27]. The same experimental procedure described above to measure the effects of citrus flavanones (100–300 *μ*M) on fatty acid oxidation was conducted. The data were expressed as nmol of O_2_ consumed per min per mg of mitochondrial protein. 

### 2.7. Peroxisomal Fatty Acyl-CoA Oxidase Activity

The peroxisomal fatty acyl-CoA oxidase activity was measured fluorimetrically using a modification [28] of the method described by Small et al. [29]. The assay for acyl-CoA oxidase was based on the determination of H_2_O_2_ production, which was coupled to the oxidation of 2′,7′-dichlorofluorescein diacetate (DCFH-DA) into a highly fluorescent compound (2′,7′-dichlorofluorescein (DCF)) in a reaction catalysed by exogenous peroxidase. The enzyme activity was monitored in real time by recording the variation in fluorescence. After the addition of the peroxisome-enriched fraction (0.3-0.4 mg protein/mL), the reaction was started by addition of the substrate palmitoyl-CoA (to a final concentration of 30 *μ*M). Citrus flavanones (25 or 200 *μ*M) were added to the incubation medium as a solution in dimethylformamide (0.1 M). In the control experiments the solvent was included in the reaction medium instead of the citrus flavanones. The increase in fluorescence (excitation, 503; emission, 529 nm) was recorded over a period of 10 minutes, and the activity of fatty acid acyl-CoA oxidase was expressed as nmol of DCF produced per min per mg of peroxisomal protein. The rates of H_2_O_2_ production were calculated from the linear regression analysis of the curves after subtracting the values of the blank curves.

### 2.8. Protein Determination

The protein contents of the mitochondrial and peroxisomal suspensions were measured as described by Lowry et al. [30], using the Folin phenol reagent and bovine serum albumin as a standard. 

### 2.9. D-*β*-Hydroxybutyrate Dehydrogenase Activity

The effect of citrus flavanones on the activity of D-*β*-hydroxybutyrate dehydrogenase from *Pseudomonas lemoignei* was assayed according to Bergmeyer [31]. The activity was measured by following the reduction of NAD^+^ at 340 nm using a spectrophotometer. In a 1.0 mL reaction mix, the final concentrations were 147 mM tris(hydroxymethyl)aminomethane (Tris, pH 8.0), 29 mM D-*β*-hydroxybutyrate, 1.93 mM NAD^+^, and 0.03 units *β*-hydroxybutyrate dehydrogenase. Citrus flavanones (50–200 *μ*M) were added to the incubation medium dissolved in dimethylformamide (0.1 M). In all assays, the enzyme plus citrus flavanones were preincubated together at room temperature (25°C) for 2 minutes, and the reaction was started by the addition of D-*β*-hydroxybutyrate. The increase in absorbance at 340 nm resulting from NADH formation was measured and expressed as nmol/min. Interference by citrus flavanones (absorbance at 340 nm) was excluded by running blanks. Control experiments were performed to exclude solvent effects.

### 2.10. Measurement of NADH Oxidation in a Cell-Free System

The effect of citrus flavanones on NADH oxidation in a cell-free system was measured according to the method of Chan et al. [17]. The reaction mixtures contained 0.1 M tris-HCl/1.0 mM EDTA buffer (pH 7.4), H_2_O_2_ (25 *μ*M), NADH (200 *μ*M), and horseradish peroxidase (HRP) type VI-A (0.1 *μ*M). Hesperidin and hesperetin (5–50 *μ*M) and naringenin (0.5–1.2 *μ*M) were added to the incubation medium dissolved in dimethylformamide (0.1 M). Reactions were started by the addition of H_2_O_2_ (25 *μ*M), and the oxidation of NADH was followed at 340 nm using a spectrophotometer. Control experiments were performed to exclude solvent effects.

### 2.11. Treatment of Data

Data are shown as the means ± standard errors. The statistical significance of the differences between parameters obtained in the experiments was evaluated using Student's *t*-test or Newman-Keuls test according to the context. The results are discussed in the text using *P* values, where *P* < 0.05 was the criterion used for significance. The ID_50_ were computed by numerical interpolation using a cubic spline function. Statistical analyses were performed using Graphic Pad Prism software version 3.0.

## 3. Results 

### 3.1. The Effects of Citrus Flavanones on Oxygen Uptake, ^14^CO_**2**_ Production, and Ketogenesis from Exogenous Octanoate and Endogenous Sources

**Figure 2:**
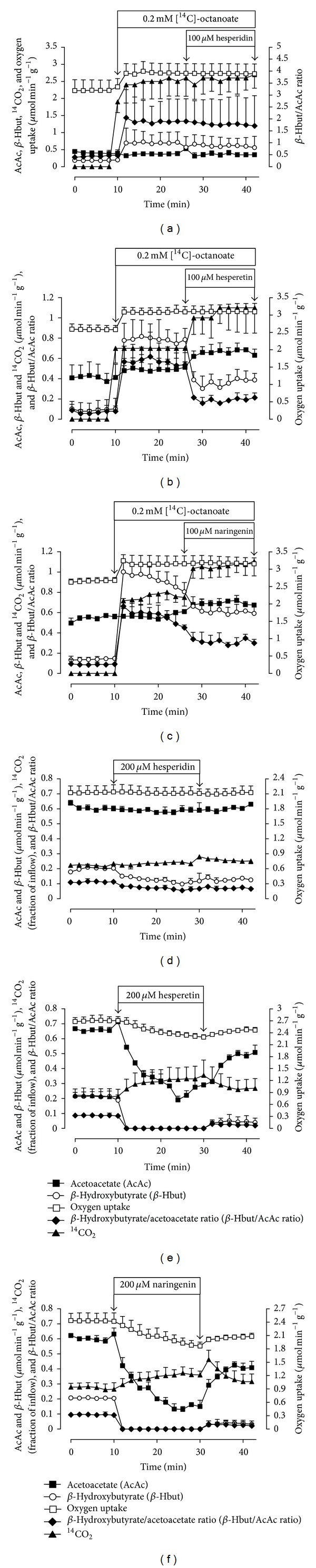
Comparison of the changes caused by hesperidin, hesperetin, and naringenin on the metabolic fluxes from exogenous octanoate ((a), (b), and (c)) and from endogenous fatty acids ((d), (e), and (f)) in perfused livers isolated from fasted rats. The effluent perfusate was analysed for acetoacetate (AcAc), *β*-hydroxybutyrate (*β*-Hbut), and ^14^CO_2_. Oxygen uptake was followed polarographically. Values are expressed as the means ± standard errors of the mean of three animals (three liver perfusion experiments) for each experimental condition.

**Figure 3:**
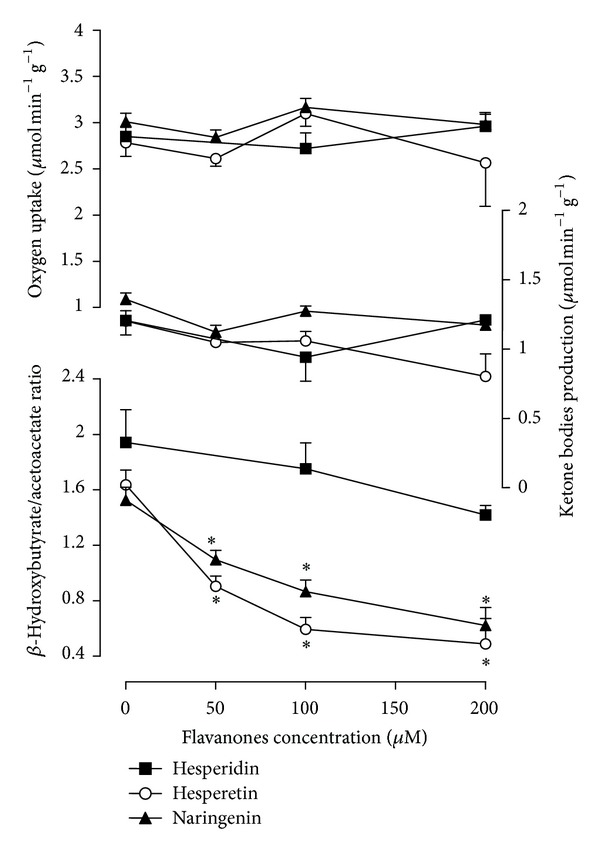
Concentration-dependent effects of hesperidin, hesperetin, and naringenin on octanoate metabolism. Data were obtained from experiments similar to those illustrated in Figure 2. Values in the absence of citrus flavanones (control values) are the mean values before the onset of citrus flavanones infusion. Values in the presence of citrus flavanones were computed at the end of the citrus flavanone infusion. Each experimental point is the mean ± standard error of three animals (three liver perfusion experiments) for each experimental condition. Asterisks indicate a statistically significant difference compared to the control condition, as revealed by variance analysis with post hoc Newman-Keuls testing (*P < 0.05).

To investigate the effects of citrus flavanones on octanoate metabolism, 0.2 mM octanoate and tracer amounts of [1-^14^C]octanoate (0.01 *μ*Ci/mL) were simultaneously infused into isolated perfused rat liver (10–42 minutes). The production of ^14^CO_2_ was measured, along with oxygen consumption and production of ketone bodies (*β*-hydroxybutyrate and acetoacetate). All these parameters are related to fatty acid transformation. Figures 2(a), 2(b), and 2(c) illustrate the experimental protocol and show the time courses of the changes caused by 100 *μ*M hesperidin, hesperetin, and naringenin. The livers responded rapidly to octanoate infusion, with clear signs that *β*-oxidation was enhanced. With the exception of acetoacetate production, which was slightly or not at all affected by octanoate, all of the parameters reached new steady states. Because the increase in *β*-hydroxybutyrate was higher than the increase in acetoacetate, there was a substantial increase in the *β*-hydroxybutyrate/acetoacetate ratio. Citrus flavanones (100 *μ*M) were introduced 16 minutes after starting the infusion of octanoate (at 26 minutes in the time scale of Figures 2(a), 2(b), and 2(c)). Naringenin and hesperetin caused decreases in *β*-hydroxybutyrate production (by 32.1% and 49.3%, resp., *P* < 0.05) and increased acetoacetate production (by 17.9% and 35.9%, resp., *P* < 0.05). Oxygen consumption remained unaltered in the presence of the flavanones. The productions of ^14^CO_2_ were increased by 39.0% (*P* < 0.05) and 49.3% (*P* < 0.05) by naringenin and hesperetin, respectively. Naringenin and hesperetin clearly decreased the *β*-hydroxybutyrate/acetoacetate ratio. Experiments such as those illustrated in Figures 2(a), 2(b), and 2(c), but without ^14^CO_2_ measurements, were repeated with 50 and 200 *μ*M hesperetin and naringenin and with 200 *μ*M hesperidin to establish concentration dependences for the effects. The changes in the rates of ketogenesis (for *β*-hydroxybutyrate + acetoacetate) in the *β*-hydroxybutyrate/acetoacetate ratio and in the rates of oxygen uptake are summarised in Figure 3. The values at zero citrus flavanone concentration correspond to the mean values of the metabolic fluxes just before the onset of citrus flavanone infusion (i.e., the basal rates measured at 26 minutes of perfusion time); the rates in the presence of citrus flavanones were the values evaluated at 42 minutes of perfusion time. Hesperetin and naringenin had no significant effect on ketogenesis or oxygen uptake. The most pronounced effect was that on the *β*-hydroxybutyrate/acetoacetate ratio, which was reduced in a concentration dependent manner, decreasing from 1.63 ± 0.10 to 0.59 ± 0.08 (*P* < 0.05) at 100 *μ*M hesperetin and from 1.52 ± 0.09 to 0.86 ± 0.08 (*P* < 0.05) at 100 *μ*M naringenin. Half-maximal reductions are expected at concentrations of 58.99 ± 6.50 hesperetin and 136.70 ± 5.98 *μ*M naringenin, as revealed by numerical interpolation. As shown in Figures 2(a), 2(b), 2(c), and 3, the oxidation of exogenous octanoate was not significantly modified by hesperidin.

Substrate-free perfused livers from fasted rats are entirely dependent on oxidation of endogenous fatty acids [32]. Due to high rates of fatty acid oxidation, the ketogenic activity of such livers is also pronounced. Because of the results obtained in the present work with an exogenous ketogenic substrate, the medium-chain fatty acid octanoate, the question arises whether the citrus flavanones hesperetin and naringenin also affect ketogenic activity that depends solely on endogenous substrates. The results of experiments performed under these conditions are shown in Figures 2(d), 2(e), and 2(f), which represents the rates of oxygen uptake, ketogenesis (*β*-hydroxybutyrate and acetoacetate production), ^14^CO_2_ production, and the *β*-hydroxybutyrate/acetoacetate ratio in experiments in which 200 *μ*M citrus flavanone (hesperidin, hesperetin, or naringenin) was infused for 20 minutes (10–30 minutes). Tracer amounts of [1-^14^C]octanoate (0.01 *μ*Ci/mL) were infused from the beginning of the perfusion experiments (0–42 minutes). All metabolic fluxes responded to naringenin and hesperetin in a very similar manner but showed little or no change in response to hesperidin. While hesperetin and naringenin caused rapid and continuous decreases in the production of *β*-hydroxybutyrate (to nondetectable levels (*P* < 0.05)), the inhibition of *β*-hydroxybutyrate production in the presence of 200 *μ*M hesperidin was 47.8% (*P* < 0.05). Acetoacetate production was also inhibited by hesperetin (61.1%, *P* < 0.05) and naringenin (76.4%, *P* < 0.05). In contrast, the acetoacetate production was not altered by hesperidin. We found that the total production of ketone bodies (*β*-hydroxybutyrate plus acetoacetate) and the *β*-hydroxybutyrate/acetoacetate ratio were reduced in the presence of any of the three citrus flavanones. The *β*-hydroxybutyrate/acetoacetate ratio was reduced 46.5% (*P* < 0.05), 100% (*P* < 0.05) and 100% (*P* < 0.05) by hesperidin, hesperetin, and naringenin, respectively. The oxygen uptake was progressively decreased by hesperetin (14.5%, *P* < 0.05) and naringenin (21.9%, *P* < 0.05), but no significant alteration was observed in the presence of hesperidin. It should be noted that these effects were accompanied by a significant increase in ^14^CO_2_ production. The production of ^14^CO_2_ was increased 9.9% (*P* < 0.05), 52.5% (*P* < 0.05), and 33.2% (*P* < 0.05) by hesperidin, hesperetin, and naringenin, respectively. In general, when the infusion of citrus flavanones was interrupted, the productions of acetoacetate, *β*-hydroxybutyrate and ^14^CO_2_, and the oxygen uptake showed only partial recoveries during the subsequent 12 minutes. 

If the effects of citrus flavanones on the *β*-hydroxybutyrate/acetoacetate ratio are due to interaction of these substances with the enzyme *β*-hydroxybutyrate dehydrogenase, which interconverts *β*-hydroxybutyrate and acetoacetate, it should be possible to reproduce these effects using the isolated enzyme. However, we found that the citrus flavanones, at concentrations up to 200 *μ*M, had no direct effect on the enzyme *β*-hydroxybutyrate dehydrogenase (data not shown). 

### 3.2. The Effects of Citrus Flavanones on Oxygen Uptake by Mitochondria Oxidising Fatty Acids, NADH, and Succinate

**Figure 4:**
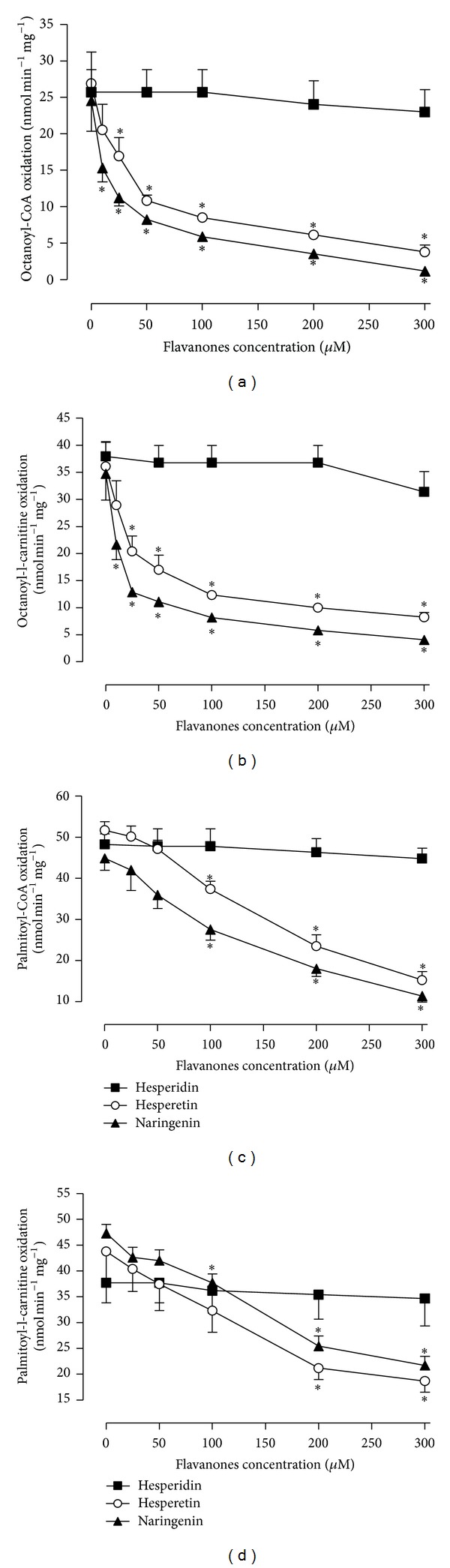
Determination of oxygen consumption by mitochondria oxidising fatty acids. The values are the means ± standard errors of the results obtained in three to six independent mitochondrial preparations, depending on the experimental series. Asterisks indicate a statistically significant difference compared to the control condition, as revealed by variance analysis with post hoc Newman-Keuls testing (*P < 0.05).

The medium-chain fatty acid octanoate was used as the oxidative substrate in some experiments with perfused liver. However, in those experiments in which the substrate-free perfused rat liver was used, respiration is mainly dependent on the oxidation of long-chain fatty acids [32]. For this reason, we also evaluated the effects of citrus flavanones (10–300 *μ*M) on oxygen uptake by mitochondria oxidising the medium-chain fatty acid octanoate and the long-chain fatty acid palmitate. These fatty acids were used as acyl-CoA derivatives (octanoyl-CoA and palmitoyl-CoA) in the presence of carnitine. Octanoyl-L-carnitine and palmitoyl-L-carnitine were also tested. As shown in Figures 4(a)–4(d), hesperetin and naringenin inhibited oxygen uptake during the oxidation of all activated fatty acids, with well-defined concentration dependencies. The ID_50_ concentrations of hesperetin for the oxidation of the activated fatty acids were octanoyl-L-carnitine, 47.0 ± 16.3 *μ*M; octanoyl-CoA, 47.9 ± 9.6 *μ*M; palmitoyl-L-carnitine, 175.7 ± 23.4 *μ*M; palmitoyl-CoA, 198.2 ± 20.7 *μ*M. The ID_50_ concentrations of naringenin for the oxidation of the activated fatty acids were octanoyl-L-carnitine, 30.8 ± 13.0 *μ*M; octanoyl-CoA, 21.3 ± 5.9; palmitoyl-L-carnitine, 225.6 ± 37.1 *μ*M; palmitoyl-CoA, 146.1 ± 16.6 *μ*M. In all cases, oxidation was not significantly altered by hesperidin.

**Figure 5:**
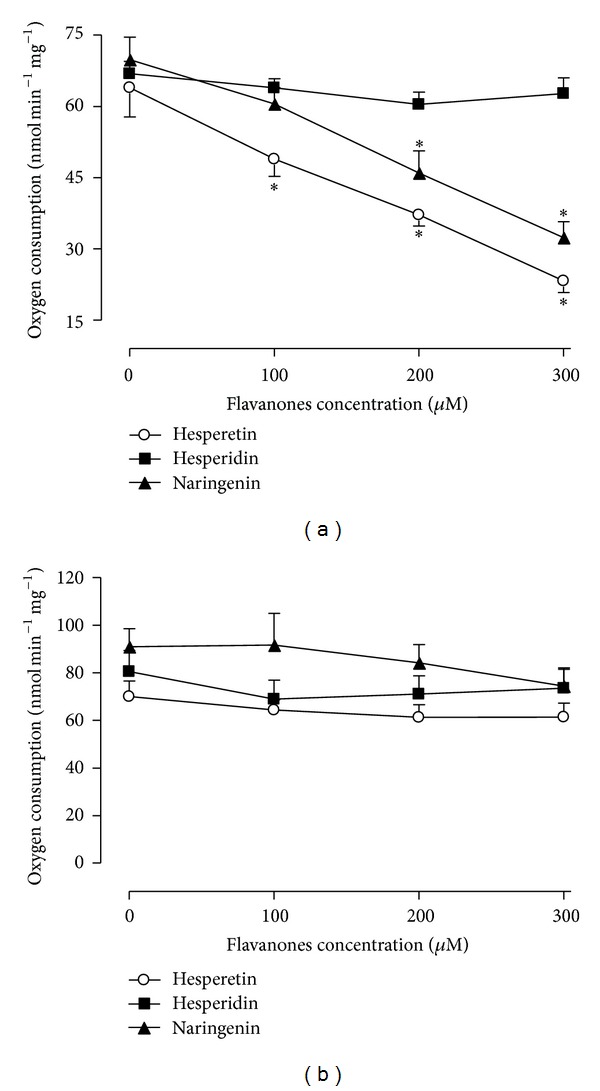
The effects of hesperidin, hesperetin and naringenin on NADH-oxidase (a) and succinate-oxidase (b) activities. The values are the means ± standard errors of the results obtained in six to seven independent mitochondrial preparations, depending on the experimental series. Asterisks indicate a statistically significant difference compared to the control condition, as revealed by variance analysis with post hoc Newman-Keuls testing (*P < 0.05).

The similar effects of hesperetin and naringenin on oxygen uptake observed in isolated rat liver mitochondria oxidising medium-chain and long-chain fatty acids raises the question of whether this effect is dependent on a direct action of citrus flavanones on mitochondrial electron flow. A way to evaluate this possibility is measurement of the respiratory activity in freeze/thaw-disrupted rat liver mitochondria using NADH (NADH-oxidase activity) or succinate (succinate-oxidase activity) as substrates for complex I and II, respectively. The illustration of Figure 5 shows that in the concentration range between 100 and 300 *μ*M, hesperetin and naringenin progressively inhibited NADH-oxidase but not succinate-oxidase activity. The ID_50_ concentrations of hesperetin and naringenin were 238.4 ± 22.19 *μ*M and 265.5 ± 23.47 *μ*M, respectively. Hesperidin had no significant effect on mitochondrial membrane-bound enzymatic activities. 

### 3.3. The Effects of Citrus Flavanones on Peroxisomal Fatty Acyl-CoA Oxidase Activity

Citrus flavanones may also interfere with peroxisomal *β*-oxidation, which supplies chain-shortened products for the complete mitochondrial fatty acid *β*-oxidation. To assess this possibility, the effects of two concentrations (25 and 200 *μ*M) of citrus flavanones on the peroxisomal acyl-CoA oxidase activity were measured in preparations containing liver peroxisome-enriched fractions. The results demonstrated that the activity of oxidising palmitoyl-CoA, which is dependent on peroxisomal fatty acyl-CoA oxidase activity, was unaffected by citrus flavanones at concentrations of up to 200 *μ*M (data not shown). 

### 3.4. The Effects of Citrus Flavanones on the Catalytic Oxidation of NADH

**Figure 6:**
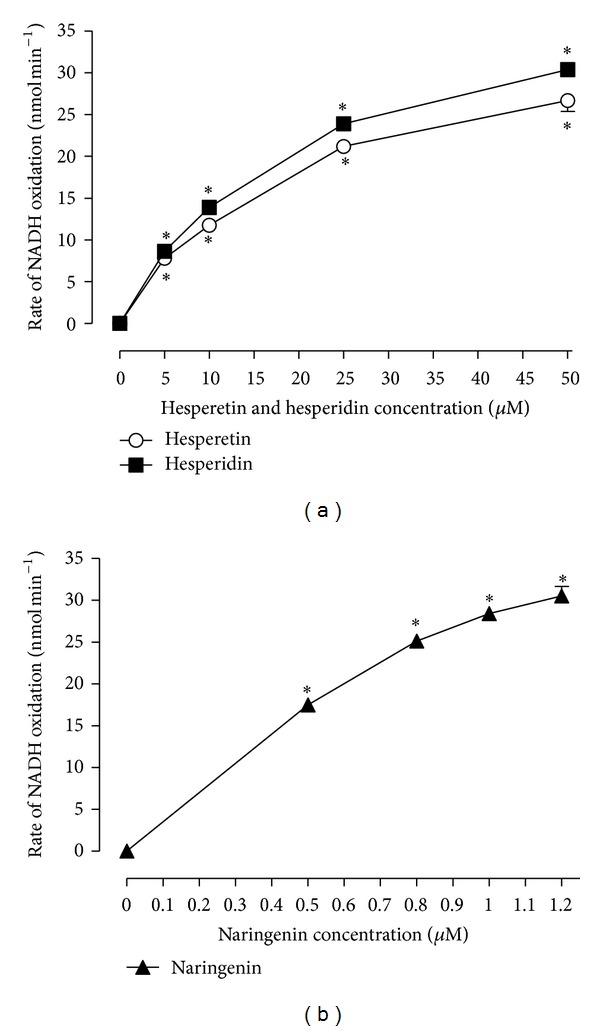
(a) and (b) show the dependence of the rate of peroxidase-catalysed NADH oxidation on the concentrations of the flavanones hesperidin, hesperetin, and naringenin. Values represent the means ± standard errors of three separate experiments. Asterisks indicate a statistically significant difference compared to the control condition, as revealed by variance analysis with post hoc Newman-Keuls testing (*P < 0.05).

Hesperidin, hesperetin, and naringenin promoted NADH oxidation in the presence of peroxidase and catalytic amounts of H_2_O_2_ (Figure 6). This action is consistent with previous reports indicating that these substances are able to form prooxidant metabolites that cooxidise NADH [17].

## 4. Discussion

Confirming the central hypothesis raised above, we found that the citrus flavanones hesperidin, hesperetin, and naringenin are able to induce a more oxidised state in liver cells by altering several parameters related to hepatic fatty acid oxidation, namely, oxygen uptake, citric acid cycle activity, and ketogenesis. This oxidative state was revealed by the decrease in the *β*-hydroxybutyrate/acetoacetate ratio in the presence of citrus flavanones, indicating a shift in the mitochondrial redox state to a more oxidised condition [33]. The *β*-hydroxybutyrate/acetoacetate ratio in the perfused liver is an indicator for the mitochondrial NADH/NAD^+^ ratio because the enzyme *β*-hydroxybutyrate dehydrogenase is present solely in the mitochondria and also because it operates under near-equilibrium conditions [12]. Furthermore, the stimulation of ^14^CO_2_ production indicated that the activity of the citric acid cycle was increased in the perfused livers. Under normal conditions, the rate of the citric acid cycle is strictly dependent on NADH reoxidation via the mitochondrial respiratory chain. However, a parallel increase in the oxygen consumption by the livers was not observed. Thus, a diversion of the NADH generated in the citric acid cycle from the respiratory chain to another oxidative reaction was raised as a possible explanation for such a phenomenon. The mechanisms by which citrus flavanones exert this effect are relatively complex and likely involve biotransformation reactions that occur in the liver, especially intracellular formation of phenoxyl radicals that can oxidise NADH in the presence of peroxidases and hydrogen peroxide [17, 18]. The fact that phenoxyl radicals have been implicated in the initiation of atherosclerosis and carcinogenesis by xenobiotic phenolic metabolites must be taken into consideration [17, 34–36].

As demonstrated in previous reports [17] and confirmed in the present work (Figure 6), the citrus flavanones have a strong ability to oxidise NADH in the presence of horseradish peroxidase (HRP) and hydrogen peroxide even when low flavanone concentrations are used. It is well known that hydrogen peroxide is always present in the liver, which possesses many enzymatic systems that are capable of producing free radicals from phenolic or polyphenolic compounds, so that it is quite possible that the same phenomena occur in the intact perfused liver [37–40]. It is unlikely that the citrus flavanones act directly on the mitochondrial enzyme *β*-hydroxybutyrate dehydrogenase because even at 200 *μ*M they did not affect the activity of this enzyme in kinetic enzyme assays.

The effect of citrus flavonoids on the NADH/NAD^+^ redox potential was accompanied by a number of other effects. In the isolated perfused rat liver, citrus flavonoids clearly inhibited ketogenesis from endogenous fatty acids, which is mainly dependent on oxidation of the long-chain fatty acids [32], but had little effect on ketogenesis from the exogenously supplied medium-chain fatty acid octanoate. Paradoxically, hesperetin and naringenin (but not hesperidin) were able to inhibit octanoate and palmitate oxidations in isolated mitochondria regardless of their chemical compositions (acyl-CoA or acyl-carnitine derivatives). Thus, the inhibition of fatty acid oxidation in isolated mitochondria likely occurs at some common point in the oxidation process of these compounds. Impairments in the respiratory chain at complex I can lead to secondary inhibition of oxidation of the fatty acids derivatives whose oxidation depends on the direct action of NAD^+^-dependent dehydrogenases [41–43]. Thereby, it is highly probable that naringenin and hesperetin inhibit the mitochondrial *β*-oxidation of fatty acids in isolated mitochondria by means of an inhibitory effect on the electron flow at complex I of the respiratory chain. It is unlikely, however, that the inhibition of ketogenesis observed in the substrate-free perfused liver is due to primary action of citrus flavanones on mitochondrial electron flow. If citrus flavanones were acting directly and solely on the respiratory chain, the *β*-hydroxybutyrate/acetoacetate ratio would be increased rather than decreased, as in similar perfusion experiments using isosteviol, an inhibitor of the respiratory chain [44]. 

The inhibitory actions of naringenin and hesperetin on ketone body production, oxygen uptake, and citric acid cycle stimulation in perfused livers are associated with the prooxidant effects of these flavanones. As previously demonstrated in experiments performed with isolated mitochondria [45], ketogenesis decreases with lower NADH/NAD^+^ ratios because the near-equilibrium of 3-hydroxyacyl-CoA dehydrogenase is shifted toward acetoacetyl-CoA, which inhibits acetyl-CoA acetyltransferase. The same phenomenon, however, favours the citric acid cycle, mainly because a less reduced state of the NADH-NAD^+^ pair shifts the near equilibrium catalysed by L-malate dehydrogenase in the direction of oxaloacetate, the acceptor of acetyl-CoA, thus favouring the action of citrate synthase. Oxygen uptake inhibition most likely occurs because the NADH concentration in the mitochondria is diminished. In addition, it is improbable that the effects of the citrus flavanones found in the present work reflect an action on peroxisomal *β*-oxidation because peroxisomal fatty acyl-CoA oxidase activity was unaffected by citrus flavanones at concentrations of up to 200 *μ*M.

Another interesting point is the difference between the effects of citrus flavanones obtained in the presence of octanoate and in the substrate-free perfused liver. Though prooxidant effect of hesperetin and naringenin are present in both cases, as shown by reductions in the mitochondrial NADH/NAD^+^ ratios, these effects were most prominent in the substrate-free perfused liver. This difference can be explained by the higher reducing power established by the introduction of 0.2 mM octanoate. If one looks at the absolute values of the *β*-hydroxybutyrate/acetoacetate ratios in the absence of citrus flavanones, it is clear that the maximal value in the presence of octanoate is higher than the value in its absence (i.e., in the substrate-free perfused liver). This is due to the enormous increases in the mitochondrial NADH/NAD^+^ ratio that are produced by the flow of reducing equivalents from exogenous octanoate. In this case, the competition for NADH between the respiratory chain and the flavanone metabolites (phenoxyl radicals) is minimised, favouring the respiratory chain. This assumption is consistent with the observation that neither oxygen uptake nor ketone body production was significantly affected by treatment with the citrus flavanones in the presence of exogenous octanoate.

Though hesperidin strongly oxidises NADH in the presence of horseradish peroxidase and hydrogen peroxide, as revealed in an *in vitro *incubation system, this flavanone did not directly affect the mitochondrial or peroxisomal activities. It exerted only little to no metabolic effects in the liver, even in the substrate-free perfused liver, most likely because of its low lipophilicity and interactions with membranes. As previously demonstrated in a biophysical study, hesperetin (i.e., the aglycone form) interacts with membranes more strongly than hesperidin (the glycoside form) does. Hesperidin, due to its rutinoside moiety, is located at the level of the polar headgroup, whereas hesperetin interacts better with acyl chains and adopts a more planar conformation [46]. Indeed, this difference could impair hesperidin transport across cellular and organelle membranes, thus decreasing its prooxidant action within hepatocytes. Our results also indicated that although hesperetin and naringenin differ in the substituents of the B-ring (hesperetin has an additional methoxy functional group (–OCH_3_) at the paraposition and its hydroxyl group is substituted at the metaposition), these differences did not have a great influence on their metabolic effects in the liver. Moreover, in general terms, comparison of the effects of the citrus flavanones (with the exception of hesperidin) revealed in the present work and those reported for the flavonols quercetin and fisetin [12, 13] show that the structural differences between these specific citrus flavonoids does not significantly alter their metabolic effects in the liver.

It is noteworthy that the concentration range of citrus flavanones used in the present study is in line with the concentrations used in cell culture systems [47] and in animals fed diets supplemented with citrus flavanones [48]. In addition, it is also known that the portal concentration of xenobiotics can achieve levels much higher than in the systemic circulation and also that the bile concentration can reach values 100 times higher than the plasma [49, 50]. Thus, it is highly probable that the portal concentrations used in the aforementioned studies reach values that are similar to those used in this work.

## 5. Conclusion

In conclusion, at least in isolated perfused rat liver, the prooxidant effects of the citrus flavanones hesperetin, naringenin and, to a lesser extent, hesperidin seem to predominate over their antioxidant effects [1], affecting the liver metabolism in several ways. To exert these prooxidant effects, the citrus flavanones most likely require cellular peroxidases, which in the presence of hydrogen peroxide, oxidise phenols to phenoxyl radicals [17, 18]. The information provided in this study counteracts the widely disseminated idea that citrus flavonoids, acting as antioxidants, exhibit beneficial effects in inflammation, atherosclerosis, and cancer [2–4]. While the effects of hesperidin, hesperetin, and naringenin on liver metabolism are certainly very complex, and while it is difficult to predict the net effects that the substances will exert *in vivo*, these results aim to improve our understanding of the mode of action of citrus flavanones on mammalian cells, particularly liver cells. However, since citrus flavanones are intensively metabolized when orally administered [51, 52], the possibility that some of their metabolites could be responsible for part of their effects (*in vivo*) cannot be excluded. Thus, despite the findings presented, further experimental investigations are necessary to provide detailed information about the effects of citrus flavanones metabolites and other citrus flavanones concentrations on hepatic metabolism and the physiological significance of such effects on the whole organism.

## References

[1] di Majo D, Giammanco M, la Guardia M, Tripoli E, Giammanco S, Finotti E (2005). Flavanones in Citrus fruit: structure-antioxidant activity relationships. *Food Research International*.

[2] Wilcox LJ, Borradaile NM, Huff MW (1999). Antiatherogenic properties of naringenin, a citrus flavonoid. *Cardiovascular Drug Reviews*.

[3] da Silva Emim JA, Oliveira AS, Lapa AJ (1994). Pharmacological evaluation of the anti-inflammatory activity of a citrus bioflavonoid, hesperidin, and the isoflavonoids, duartin and claussequinone, in rats and mice. *The Journal of Pharmacy and Pharmacology*.

[4] Manthey JA, Guthrie N, Grohmann K (2001). Biological properties of citrus flavonoids pertaining to cancer and inflammation. *Current Medicinal Chemistry*.

[5] Heim KE, Tagliaferro AR, Bobilya DJ (2002). Flavonoid antioxidants: chemistry, metabolism and structure-activity relationships. *The Journal of Nutritional Biochemistry*.

[6] Bors W, Heller W, Michel C, Saran M (1990). Flavonoids as antioxidants: determination of radical-scavenging efficiencies. *Methods in Enzymology*.

[7] de Groot H, Rauen U (1998). Tissue injury by reactive oxygen species and the protective effects of flavonoids. *Fundamental and Clinical Pharmacology*.

[8] Bors W, Michel C, Stettmaier K (2001). Structure-activity relationships governing antioxidant capacities of plant polyphenols. *Methods in Enzymology*.

[9] Sengupta B, Banerjee A, Sengupta PK (2004). Investigations on the binding and antioxidant properties of the plant flavonoid fisetin in model biomembranes. *FEBS Letters*.

[10] Constantin J, Bracht A, Sing K, Govil JN (2008). Quercetin, mechanisms of anti and prooxidant activities. *Recent Progress in Medicinal Plants, Volume 21: Phytopharmacology and Therapeutic Values III*.

[11] Cao G, Sofic E, Prior RL (1997). Antioxidant and prooxidant behavior of flavonoids: structure-activity relationships. *Free Radical Biology and Medicine*.

[12] Buss GD, Constantin J, de Lima LCN (2005). The action of quercetin on the mitochondrial NADH to NAD+ ratio in the isolated perfused rat liver. *Planta Medica*.

[13] Constantin RP, Constantin J, Pagadigorria CLS (2011). Prooxidant activity of fisetin: effects on energy metabolism in the rat liver. *Journal of Biochemical and Molecular Toxicology*.

[14] Gasparin FRS, Salgueiro-Pagadigorria CL, Bracht L, Ishii-Iwamoto EL, Bracht A, Constantin J (2003). Action of quercetin on glycogen catabolism in the rat liver. *Xenobiotica*.

[15] Gasparin FRS, Spitzner FL, Ishii-Iwamoto EL, Bracht A, Constantin J (2003). Actions of quercetin on gluconeogenesis and glycolysis in rat liver. *Xenobiotica*.

[16] Constantin RP, Constantin J, Pagadigorria CLS (2010). The actions of fisetin on glucose metabolism in the rat liver. *Cell Biochemistry and Function*.

[17] Chan T, Galati G, O’Brien PJ (1999). Oxygen activation during peroxidase catalysed metabolism of flavones or flavanones. *Chemico-Biological Interactions*.

[18] Galati G, Sabzevari O, Wilson JX, O’Brien PJ (2002). Prooxidant activity and cellular effects of the phenoxyl radicals of dietary flavonoids and other polyphenolics. *Toxicology*.

[19] Scholz R, Bücher T, Chance B, Estabrook RW, Williamson JR (1965). Hemoglobin-free perfusion of rat liver. *Control of Energy Metabolism*.

[20] Soboll S, Heldt HW, Scholz R (1981). Changes in the subcellular distribution of metabolites due to ethanol oxidation in perfused rat liver. *Hoppe-Seyler’s Zeitschrift fur Physiologische Chemie*.

[21] Mellanby J, Williamson DH, Bergmeyer HU (1974). Acetoacetate. *Methods of Enzymatic Analysis*.

[22] Clark LC (1956). Monitoring and control of blood O_2_ tension. *Transactions American Society For Artificial Internal Organs*.

[23] Scholz R, Olson MS, Schwab AJ (1978). The effect of fatty acids on the regulation of pyruvate dehydrogenase in perfused rat liver. *European Journal of Biochemistry*.

[24] Voss DO, Campello AP, Bacila M (1961). The respiratory chain and the oxidative phosphorylation of rat brain mitochondria. *Biochemical and Biophysical Research Communications*.

[25] Natarajan SK, Eapen CE, Pullimood AB, Balasubramanian KA (2006). Oxidative stress in experimental liver microvesicular steatosis: role of mitochondria and peroxisomes. *Journal of Gastroenterology and Hepatology*.

[26] Garland PB, Shepherd D, Nicholls DG, Yates DW, Light PA, Lowestein JM (1969). Interactions between fatty acid oxidation and the tricarboxylic acid cycle. *Citric Acid Cycle*.

[27] Singer TP (1974). Determination of the activity of succinate, NADH, choline, and alpha-glycerophosphate dehydrogenases. *Methods of Biochemical Analysis*.

[28] Taguchi H, Ogura Y, Takanashi T, Hashizoe M, Honda Y (1996). In vivo quantitation of peroxides in the vitreous humor by fluorophotometry. *Investigative Ophthalmology and Visual Science*.

[29] Small GM, Burdett K, Connock MJ (1985). A sensitive spectrophotometric assay for peroxisomal acyl-CoA oxidase. *The Biochemical Journal*.

[30] Lowry OH, Rosebrough NJ, Farr AL, Randall RJ (1951). Protein measurement with the Folin phenol reagent. *The Journal of Biological Chemistry*.

[31] Bergmeyer HU, Bergmeyer HU (1974). 3-hydroxybutyrate dehydrogenase from rhodopseudomonas spheroids. *Methods of Enzymatic Analysis*.

[32] Soboll S, Scholz R, Heldt HW (1978). Subcellular metabolite concentrations. Dependence of mitochondrial and cytosolic ATP systems on the metabolic state of perfused rat liver. *European Journal of Biochemistry*.

[33] Veech RL, Raijman L, Krebs HA (1970). Equilibrium relations between the cytoplasmic adenine nucleotide system and nicotinamide-adenine nucleotide system in rat liver. *Biochemical Journal*.

[34] Subrahmanyam VV, Ross D, Eastmond DA, Smith MT (1991). Potential role of free radicals in benzene-induced myelotoxicity and leukemia. *Free Radical Biology and Medicine*.

[35] Savenkova MI, Mueller DM, Heinecke JW (1994). Tyrosyl radical generated by myeloperoxidase is a physiological catalyst for the initiation of lipid peroxidation in low density lipoprotein. *Journal of Biological Chemistry*.

[36] Daugherty A, Dunn JL, Rateri DL, Heinecke JW (1994). Myeloperoxidase, a catalyst for lipoprotein oxidation, is expressed in human atherosclerotic lesions. *Journal of Clinical Investigation*.

[37] Aebi H, Bergmeyer HU (1974). Catalase. *Methods of Enzymatic Analysis*.

[38] Pütter J, Bergmeyer HU (1974). Peroxidases. *Methods of Enzymatic Analysis*.

[39] Komatsu H, Koo A, Ghadishah E (1992). Neutrophil accumulation in ischemic reperfused rat liver: evidence for a role for superoxide free radicals. *The American Journal of Physiology—Gastrointestinal and Liver Physiology*.

[40] Mason RP, Fischer V (1986). Free radicals of acetaminophen: their subsequent reactions and toxicological significance. *Federation Proceedings*.

[41] Fromenty B, Fisch C, Labbe G (1990). Amiodarone inhibits the mitochondrial *β*-oxidation and fatty acids and produces microvesicular steatosis of the liver in mice. *Journal of Pharmacology and Experimental Therapeutics*.

[42] Mannaerts GP, Debeer LJ, Thomas J, de Schepper PJ (1979). Mitochondrial and peroxisomal fatty acid oxidation in liver homogenates and isolated hepatocytes from control and clofibrate-treated rats. *Journal of Biological Chemistry*.

[43] Watmough NJ, Bindoff LA, Birch-Machin MA (1990). Impaired mitochondrial *β*-oxidation in a patient with an abnormality of the respiratory chain. Studies in skeletal muscle mitochondria. *Journal of Clinical Investigation*.

[44] Kelmer-Bracht AM, Kemmelmeier FS, Ishii-Iwamoto EL, Alvarez M, Bracht A (1985). Effect of Stevia rebaudiana natural products on cellular and sub-cellular metabolism. *Arquivos de Biologia e Tecnologia*.

[45] Stermann WH, Holze G, Seubert W, Söling HD, Seufert CD (1978). Regulation of ketogenesis at the site of acetyl-CoA acetyl transferase. *Biochemical and Clinical Aspects of Ketone Body Metabolism*.

[46] Londoño-Londoño J, Lima VRD, Jaramillo C, Creczynski-pasa T (2010). Hesperidin and hesperetin membrane interaction: understanding the role of 7-O-glycoside moiety in flavonoids. *Archives of Biochemistry and Biophysics*.

[47] Goldwasser J, Cohen PY, Yang E, Balaguer P, Yarmush ML, Nahmias Y (2010). Transcriptional regulation of human and rat hepatic lipid metabolism by the grapefruit flavonoid naringenin: role of PPAR*α*, PPAR*γ* and LXR*α*. *PLoS One*.

[48] Felgines C, Texier O, Morand C (2000). Bioavailability of the flavanone naringenin and its glycosides in rats. *American Journal of Physiology—Gastrointestinal and Liver Physiology*.

[49] Hoffman DJ, Seifert T, Borre A, Nellans HN (1995). Method to estimate the rate and extent of intestinal absorption in conscious rats using an absorption probe and portal blood sampling. *Pharmaceutical Research*.

[50] Saller R, Brignoli R, Melzer J, Meier R (2008). An updated systematic review with meta-analysis for the clinical evidence of silymarin. *Forschende Komplementarmedizin*.

[51] Matsumoto H, Ikoma Y, Sugiura M, Yano M, Hasegawa Y (2004). Identification and quantification of the conjugated metabolites derived from orally administered hesperidin in rat plasma. *Journal of Agricultural and Food Chemistry*.

[52] Mullen W, Archeveque M-A, Edwards CA, Matsumoto H, Crozier A (2008). Bioavailability and metabolism of orange juice flavanones in humans: impact of a full-fat yogurt. *Journal of Agricultural and Food Chemistry*.

